# A Fatal Case of Unnatural Labor

**Published:** 1868-01

**Authors:** W. C. Bellamy

**Affiliations:** Columbus, Georgia


### ARTICLE II.

*A Fatal Case of Unnatural Labor.—Death resulting from the base Mal-practice of an ignorant Negro Midwife.* Dr. W. C. Bellamy, Columbus, Ga.

Witnessing the horrible case, a history of which I am about to relate, the question naturally presents itself: How long will the law of the land leave the lives of valuable individuals not only in the hands of charlatans and quacks, but, also, even worse, in the hands of ignorant and illiterate negroes? I say leave lives in their hands, for it is nothing more. Is it not equivalent to sanctioning it when they do not forbid an ignorant old negro woman set herself up to practice midwifery when she knows no more about the formation of the pelvis, (not even the meaning of the word) and the functions of the womb and appendages, than she does about manufacturing a watch? And then another question here presents itself: How long will it take those, even, who pass for intelligent white men, to learn the danger of trusting their wives in the hands of such miserable creatures? You cannot find a man in the State who would give his watch to an old negro woman to repair; and yet I am sorry to say, it is but too often the case that they will allow one to deliver their wives, when they know no more about one than the other.

But to the case in point, which is one of unusual interest, because of such rare occurrence, and so severe in its consequences.

On Monday, October 14, I was called from dinner to see a negro woman in labor. On my arrival, I found her very much exhausted, with strong, rapid uterine contractions, and the left arm of the child hanging out of the vagina, the parts very much swollen, and the child firmly impacted in the inferior strait and vagina. Upon inquiry, I found she had been in labor ever since the Saturday night previous, the liquor amnii having escaped at that time, and the arm of the child *pulled out* by the old negro midwife in attendance, who professed to know all about it, and opposed calling in a physician, telling her all the time everything was progressing "*all right.*"

Finding the arm out, my first impulse was to replace it, turn and deliver the woman. But from the violent and rapid contractions, swollen parts, and impacted condition of the child, this was absolutely impossible. The patient, suffering such agony, and being so prostrated, I gave a her grain of morphine in a little brandy and water. Finding it impossible to turn the child in this condition, I thought that if the arm was disarticulated at the shoulder, I could then turn it. The child being already dead, which I have neglected before to mention, I felt no delicacy in taking off the arm, and proceeded immediately to do so. But to my disappointment, I found the difficulty not a whit removed. Discovering at this stage that some other means of delivery were necessary, and the patient anxious for rest, I gave her another dose of morphine and brandy, and went out in search of my friend and confrere, Dr. V. H. Taliaferro, for assistance, and to procure craniotomy, or some other instruments. We returned together in about three quarters of an hour, and, after consultation, sent for Dr. M. J. Moses and Dr. J. J. Mason, both for assistance and on account of the unusual interest of the case, and Dr. Edwin DeGraffenreid was also invited to witness the case. Each in turn having made an examination, several thought it possible to turn and deliver, since the arm had been disposed of and was out of the way, but every attempt at it proved utterly futile. We, therefore, decided to eviscerate the fœtus, and, if impossible to delivery, then to dissect it away. I, therefore, took a pair of craniotomy instruments and endeavored to puncture the chest, but the bulk of the child so totally filled the vagina, where it now was, I was unable to pass my hand with the instrument, to the point of puncture. I, therefore, requested Dr. Moses, who had a smaller hand, to make the puncture, which he did near the left nipple. I passed my hand and enlarged the opening, and extracted a small portion of the thoracic viscera, but the impaction was so close, I was compelled to ask the assistance of some of the other gentlemen with smaller hands. After having at last got away the thoracic viscera, the diaphragm was torn asunder and the abdominal viscera extracted, but still the child did not collapse sufficiently to come away. After various unsuccessful attempts now to deliver, we decided to decapitate the fœtus, as we could pass our fingers around the neck. We, therefore, directed with the hand a pair of long bladed bone nippers to the neck, and after several attempts, succeeded in cutting through the cervical vertebræ, though the integuments were not entirely severed. This made the neck, however, more pliant, not so stiff, and capable of being bent upon the body, and after a great deal of trouble, time, patience, and considerable force, it was drawn forth with the blunt hook. After waiting a sufficient time for nature to save me the trouble, till I saw she would not do it, I inserted my hand, (this time with great ease) and took away the placenta, which was already almost, if not entirely detached. I then, by every means I could think of endeavored to establish a proper contraction of the uterus after the delivery, but in vain, the womb seeming to have lost all power to contract, and appearing to be perfectly paralyzed; nor did there follow any discharge of lochia at all. I applied a bandage secundum artem, gave the patient a glass of brandy and water, 2 drachms of powdered ergot, made her comfortable in bed, and bade her take her rest till morning, it now being 9 o'clock at night, we having worked with her from 3 in the afternoon till that time. My professional friends retired, but I remained with her till morning, in case I might be needed. Her pulse was now very quick, but remarkably weak and thready, and instead of any hemorrhage or lochia, there was a kind of dark, sanious fluid slowly dripping away, and with a slight bubbling sound, like air passing through water, whenever she would move. There was extreme tenderness over the whole abdomen, and with this critical pulse, cold extremeties and great constipation, I feared the supervention of peritonitis, so I ordered:

Ol. Ricini, ℥ ii.

Ol. Terebinth, ʒ i.

Sig. Take at once.

The nurses told me next morning they gave it and it operated, but they were no less ignorant than indifferent, and I am not sure they told me truth. At any rate, by the middle of the afternoon of Tuesday following the delivery, the pulse had subsided somewhat, the patient was more quiet and rational, and apparently in a more encouraging condition. But from the fact that the tenderness of the peritoneum and the escape of that same dark, unhealthy looking fluid continued, and no uterine contraction, or after pains, or lochia having come on, I very seriously feared a fatal termination. Therefore, after again making various vain attempts to establish the uterine contractions and the discharge of the lochia, I gave her again another dose of morphine, and left her for a few hours. At about noon on the Wednesday following her delivery, I called on my patient, and found her evidently rapidly sinking. She was then almost in an entirely comatose state, no contractions and no lochia having as yet been established. Indeed, I considered her then beyond the reach of all human aid. I, however, endeavored to strengthen her up, and, therefore, gave her brandy and water, beef tea, chicken water, &c., and applied sinapisms to her wrists and ankles, which, however, produced no more effect than if they had been applied to a statue. Her surface, particularly the extremeties, were now cold and clammy. I ordered jugs of hot water placed around her, with a view to warming her up, but all to no purpose.

On Thursday at noon they sent for me, with delight telling me she was a great deal better. I went and found her more rational and intelligent, but—not better—and I told them it was only the bright, intelligent moments which so often precede a speedy dissolution. I saw that nothing could be done for her, and consequently left her. In the afternoon they again sent for me, telling me she was worse, and I went to her immediately, (not with the hope of accomplishing any good, but only to fulfill my duty to her,) and found her dying, and at 9 o'clock on Tuesday night, she breathed her last.

This case is recorded, not with a view of adding any improvement in the method of treating such cases, for we discovered in its treatment nothing which can be of any service in the treatment of similar cases. It was evidently a case in the management of which we could not be in the slightest guided by any knowledge heretofore gained, for it was a case entirely peculiar to itself, and in which the physician had to be guided entirely by circumstances, judgment and common sense. There is no rule laid down in the whole course of obstetrics that would apply to this case, nor could one be made. It had to be treated simply upon principles of common sense, and each symptom combatted as it presented itself. Of the five physicians present, not one of them had ever seen a case similar to it in all respects. The only wonder to me in the matter is that the patient did not die under the operation.

The child was a very large one, and the woman also was remarkably well-formed, having a very capacious pelvis and vagina, and the soft parts unusually elastic. I believe, had a physician been called at first, and before the parts became so much swollen and the child so impacted, she might have been safely delivered. The fatal termination of this case shows how reprehensible is the habit of allowing these miserable creatures to impose upon the public. They go upon the principles that the generality of cases go on naturally to a favorable termination, and yet while this is true in the main, they never know when they are going to meet with one which will not, and just so long as they are allowed to go on in this matter, experimenting with the lives of individuals, just so long are they guilty of absolute murder.

